# Supplementary figures and images for: Differential gene expression associated with a floral scent polymorphism in the evening primrose Oenothera harringtonii (Onagraceae)
Source: BMC Genomics. 2022 Feb 12;23:124. doi: 10.1186/s12864-022-08370-6 (PMC8840323; doi:10.1186/s12864-022-08370-6)

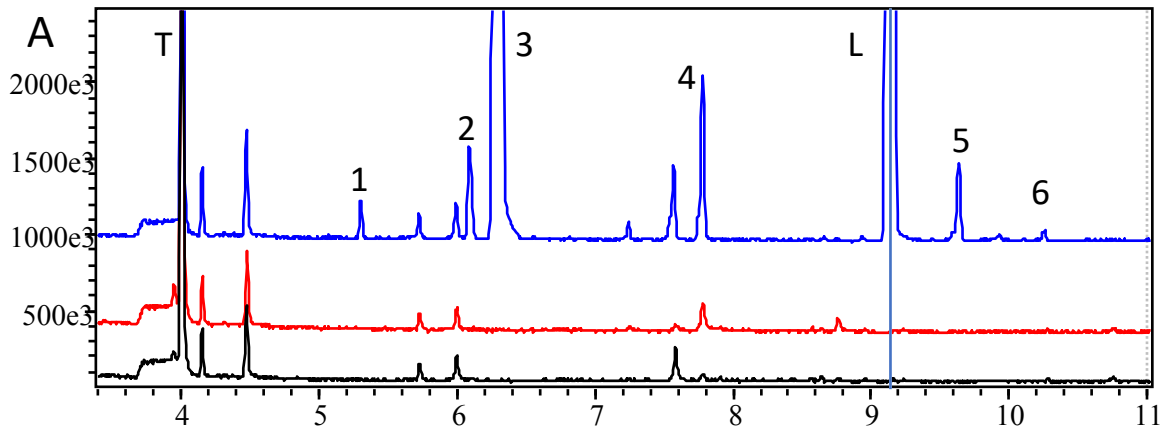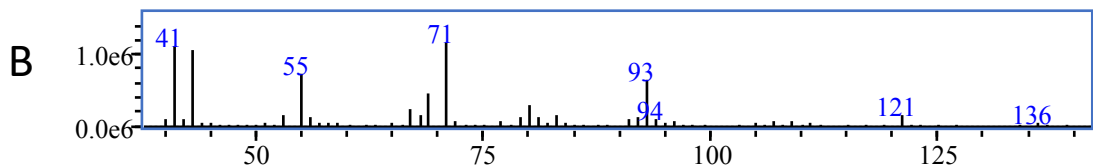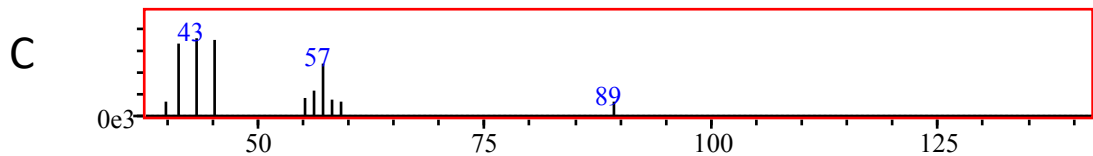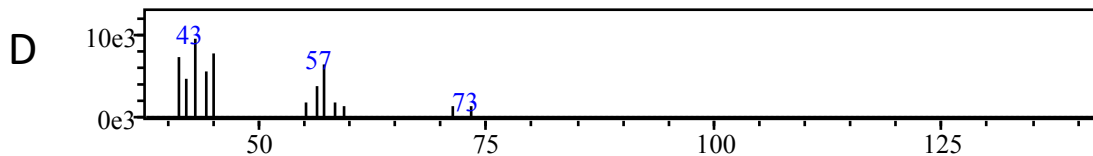

Supplement: Supplementary file 3 — Additional file 3. GC-MS evidence for the absence of constitutive volatile terpenoid emissions from the leaves of Oenothera harringtonii. Panel A: Total ion chromatograms comparing floral headspace (upper trace, blue) with two samples taken from non-blooming vegetative rosettes (middle and lower traces, red and black) of O. harringtonii from a population (Florence, Colorado, USA) producing (R)-(−)-linalool in flowers. GC traces are presented at the same scale (T = internal standard of 23.6 ng toluene) and numbered compounds represent volatile terpenoids (1 = β-myrcene, 2 = (Z)-β-ocimene, 3 = (E)-β-ocimene, 4 = ocimene epoxide, L = (R)-(−)-linalool, 5 = β-caryophyllene, 6 = α-humulene). Note that all of these peaks (including 4) are absent in the vegetative samples. Panels B, C and D show the mass spectra (m/z 40–350) at 9.15 min, the retention time for linalool under the chromatographic conditions used in this study (see methods). The correct spectrum for linalool is present only in panel B, corresponding to the floral headspace; the two vegetative samples shown have no peak at this retention time. [file 12864_2022_8370_MOESM3_ESM.pdf]
