# Supplementary material for: Differential gene expression associated with a floral scent polymorphism in the evening primrose Oenothera harringtonii (Onagraceae)
Source: BMC Genomics. 2022 Feb 12;23:124. doi: 10.1186/s12864-022-08370-6 (PMC8840323; doi:10.1186/s12864-022-08370-6)
Supplement: Supplementary file 5 — Additional file 5. Phylogeny of terpene synthases including phased alleles for the putative (R)-(−)-linalool synthase reconstructed in this study. [file 12864_2022_8370_MOESM5_ESM.pdf]

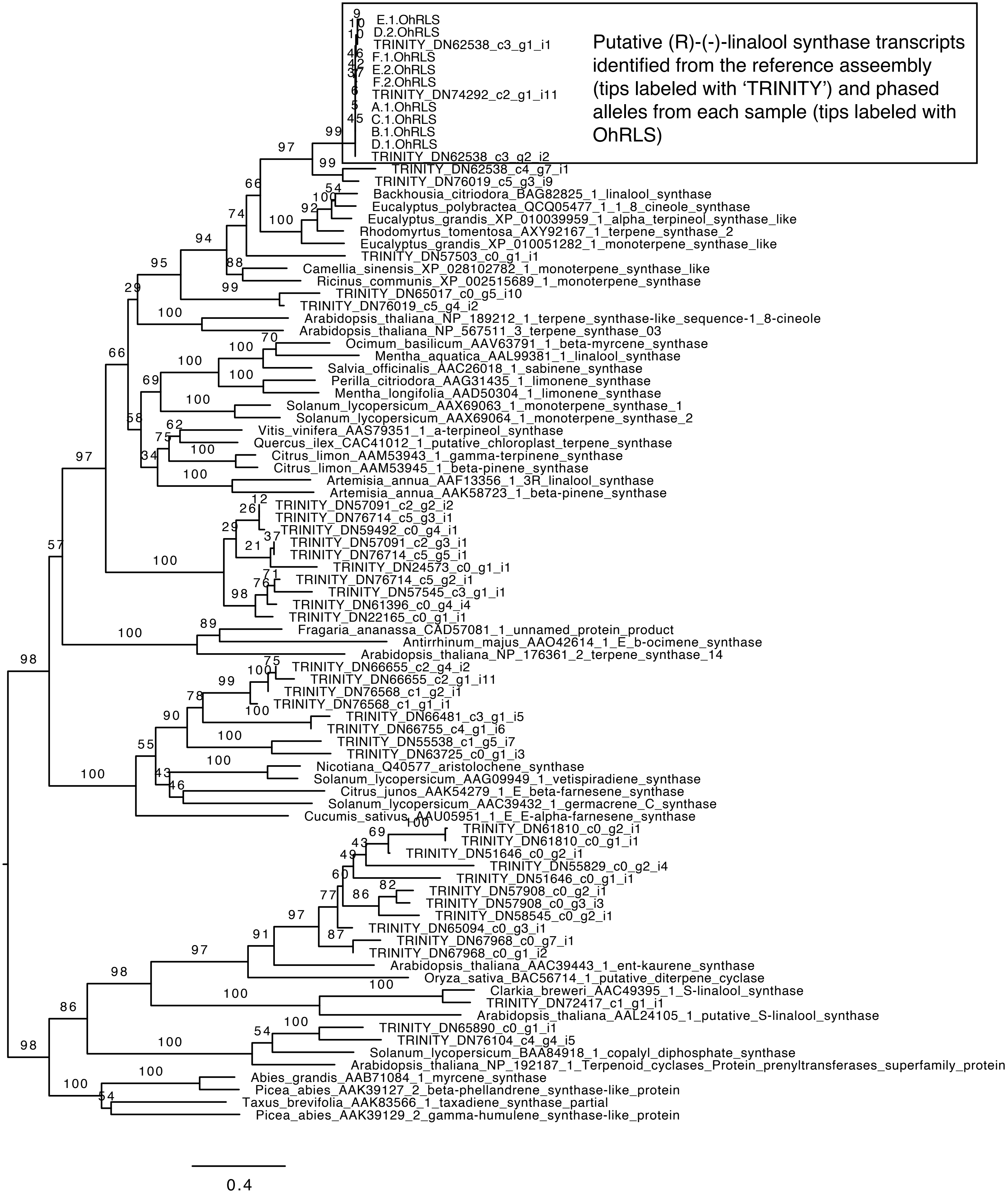
Putative (R)-(-)-linalool synthase transcripts identified from the reference asseembly (tips labeled with 'TRINITY') and phased alleles from each sample (tips labeled with OhRLS)
E.1.OhRLS
D.2.OhRLS
TRINITY_DN62538_c3_g1_i1
F.1.OhRLS
E.2.OhRLS
F.2.OhRLS
TRINITY_DN74292_c2_g1_i11
A.1.OhRLS
C.1.OhRLS
B.1.OhRLS
D.1.OhRLS
TRINITY_DN62538_c3_g2_i2
TRINITY_DN62538_c4_g7_i1
TRINITY_DN76019_c5_g3_i9
Backhousia_citriodora_BAG82825_1_linalool_synthase
Eucalyptus_polybractea_QCQ05477_1_1_8_cineole_synthase
Eucalyptus_grandis_XP_010039959_1_alpha_terpineol_synthase_like
Rhodomyrtus_tomentosa_AXY92167_1_terpene_synthase_2
Eucalyptus_grandis_XP_010051282_1_monoterpene_synthase_like
TRINITY_DN57503_c0_g1_i1
Camellia_sinensis_XP_028102782_1_monoterpene_synthase_like
Ricinus_communis_XP_002515689_1_monoterpene_synthase
TRINITY_DN65017_c0_g5_i10
TRINITY_DN76019_c5_g4_i2
Arabidopsis_thaliana_NP_189212_1_terpene_synthase-like_sequence-1_8-cineole
Arabidopsis_thaliana_NP_567511_3_terpene_synthase_03
Ocimum_basilicum_AAV63791_1_beta-myrcene_synthase
Mentha_aquatica_AAL99381_1_linalool_synthase
Salvia_officinalis_AAC26018_1_sabinene_synthase
Perilla_citriodora_AAG31435_1_limonene_synthase
Mentha_longifolia_AAD50304_1_limonene_synthase
Solanum_lycopersicum_AAX69063_1_monoterpene_synthase_1
Solanum_lycopersicum_AAX69064_1_monoterpene_synthase_2
Vitis_vinifera_AAS79351_1_a-terpineol_synthase
Quercus_ilex_CAC41012_1_putative_chloroplast_terpene_synthase
Citrus_limon_AAM53943_1_gamma-terpinene_synthase
Citrus_limon_AAM53945_1_beta-pinene_synthase
Artemisia_annua_AAF13356_1_3R_linalool_synthase
Artemisia_annua_AAK58723_1_beta-pinene_synthase
TRINITY_DN57091_c2_g2_i2
TRINITY_DN76714_c5_g3_i1
TRINITY_DN59492_c0_g4_i1
TRINITY_DN57091_c2_g3_i1
TRINITY_DN76714_c5_g5_i1
TRINITY_DN24573_c0_g1_i1
TRINITY_DN76714_c5_g2_i1
TRINITY_DN57545_c3_g1_i1
TRINITY_DN61396_c0_g4_i4
TRINITY_DN22165_c0_g1_i1
Fragaria_ananassa_CAD57081_1_unnamed_protein_product
Antirrhinum_majus_AAO42614_1_E_b-ocimene_synthase
Arabidopsis_thaliana_NP_176361_2_terpene_synthase_14
TRINITY_DN66655_c2_g4_i2
TRINITY_DN66655_c2_g1_i11
TRINITY_DN76568_c1_g2_i1
TRINITY_DN76568_c1_g1_i1
TRINITY_DN66481_c3_g1_i5
TRINITY_DN66755_c4_g1_i6
TRINITY_DN55538_c1_g5_i7
TRINITY_DN63725_c0_g1_i3
Nicotiana_Q40577_aristolochene_synthase
Solanum_lycopersicum_AAG09949_1_vetispiradiene_synthase
Citrus_junos_AAK54279_1_E_beta-farnesene_synthase
Solanum_lycopersicum_AAC39432_1_germacrene_C_synthase
Cucumis_sativus_AAU05951_1_E_E-alpha-farnesene_synthase
TRINITY_DN61810_c0_g2_i1
TRINITY_DN61810_c0_g1_i1
TRINITY_DN51646_c0_g2_i1
TRINITY_DN55829_c0_g2_i4
TRINITY_DN51646_c0_g1_i1
TRINITY_DN57908_c0_g2_i1
TRINITY_DN57908_c0_g3_i3
TRINITY_DN58545_c0_g2_i1
TRINITY_DN65094_c0_g3_i1
TRINITY_DN67968_c0_g7_i1
TRINITY_DN67968_c0_g1_i2
Arabidopsis_thaliana_AAC39443_1_ent-kaurene_synthase
Oryza_sativa_BAC56714_1_putative_diterpene_cyclase
Clarkia_breweri_AAC49395_1_S-linalool_synthase
TRINITY_DN72417_c1_g1_i1
Arabidopsis_thaliana_AAL24105_1_putative_S-linalool_synthase
TRINITY_DN65890_c0_g1_i1
TRINITY_DN76104_c4_g4_i5
Solanum_lycopersicum_BAA84918_1_copalyl_diphosphate_synthase
Arabidopsis_thaliana_NP_192187_1_Terpenoid_cyclases_Protein_prenyltransferases_superfamily_protein
Abies_grandis_AAB71084_1_myrcene_synthase
Picea_abies_AAK39127_2_beta-phellandrene_synthase-like_protein
Taxus_brevifolia_AAK83566_1_taxadiene_synthase_partial
Picea_abies_AAK39129_2_gamma-humulene_synthase-like_protein
0.4
