# Supplementary material for: Differential gene expression associated with a floral scent polymorphism in the evening primrose Oenothera harringtonii (Onagraceae)
Source: BMC Genomics. 2022 Feb 12;23:124. doi: 10.1186/s12864-022-08370-6 (PMC8840323; doi:10.1186/s12864-022-08370-6)
Supplement: Supplementary file 6 — Additional file 6. Principal components analysis of differential gene expression. [file 12864_2022_8370_MOESM6_ESM.pdf]

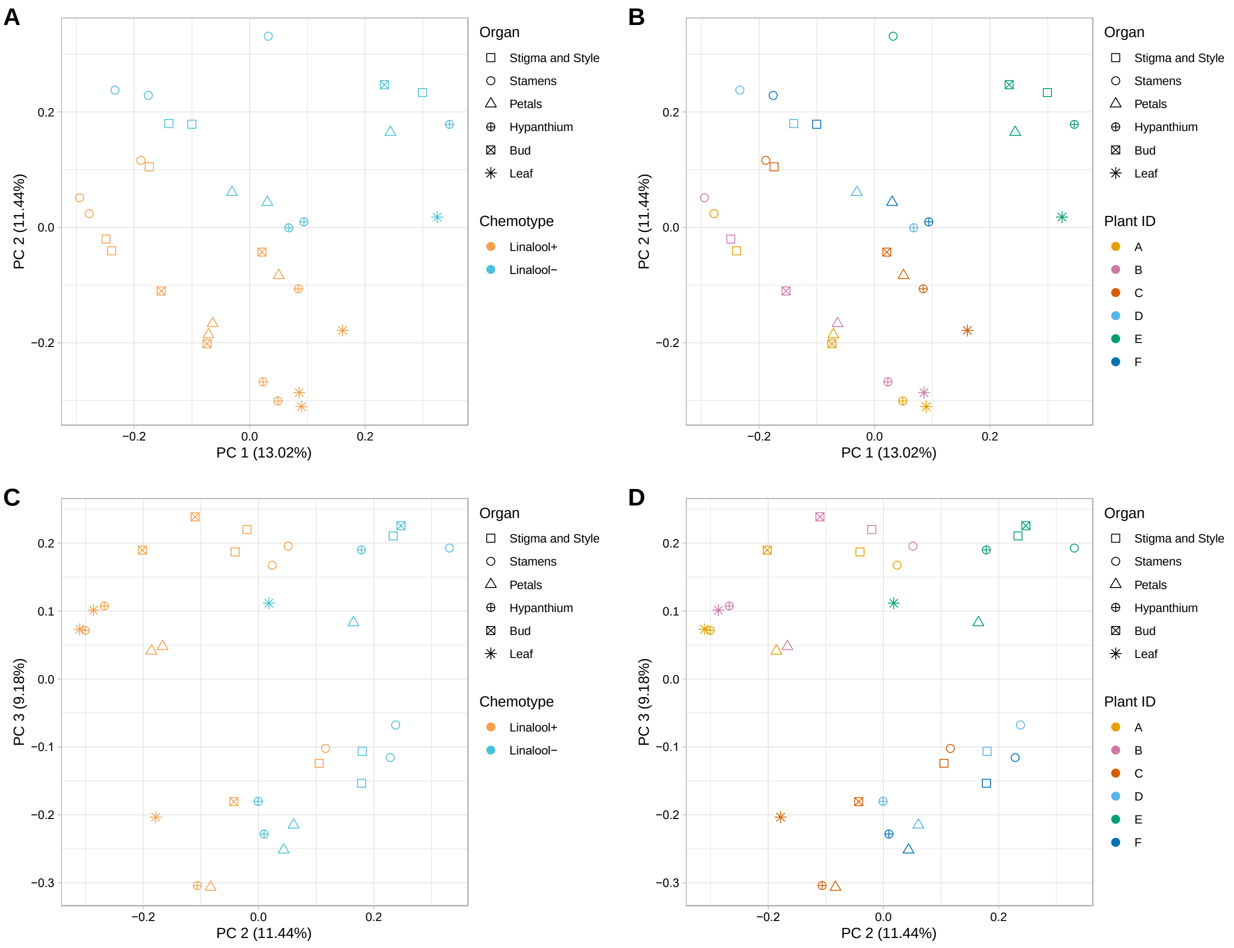

Additional File 6 (Figure): Principal Components Analysis of Differential Gene Expression. Each RNA-Seq library is represented by a symbol whose shape indicates tissue source and color indicates linalool chemotype (A and C; blue = linalool- and orange = linalool+) or individual (B and D: plants A-F). Overall, 33.64% of variation in gene expression is captured by the first three principal components. (A and B) PC 1 and 2 (C and D) PC 2 and 3.

Given that some, but not all biological replicates of the same tissue cluster together, we checked for unwanted variation using relative log expression (RLE) plots (https://journals.plos.org/plosone/article?id=10.1371/journal.pone.0191629). The RLE plot revealed two samples to be outliers: individual C stigma and style and individual C stamens. Identification of differentially expressed genes was repeated with these two samples omitted; i.e. positive stigma/style and stamens were not included in any of the comparisons made. The omission of the two samples eliminated 1056 DEGs. These DEGs primarily differed in expression among tissues (842 genes) and only 8 genes were grouped into chemotype-driven clusters 10 and 11 (none of which were annotated with functions related to floral scent). Because these outlier samples don't impact the conclusions of the study, we decided to retain these two samples in our results.
