# Supplementary material for: Differential gene expression associated with a floral scent polymorphism in the evening primrose Oenothera harringtonii (Onagraceae)
Source: BMC Genomics. 2022 Feb 12;23:124. doi: 10.1186/s12864-022-08370-6 (PMC8840323; doi:10.1186/s12864-022-08370-6)
Supplement: Supplementary file 7 — Additional file 7. Annotation of a 13,705 kb genomic contig (Contig ID 2552694) indicating the position, predicted full-length coding sequence, and predicted full-length protein sequence of the genomic homolog of the putative (R)-(−)-linalool synthase inferred from RNA-Seq data. [file 12864_2022_8370_MOESM7_ESM.doc]

# This output was generated with AUGUSTUS (version 3.3.3).

# AUGUSTUS is a gene prediction tool written by M. Stanke (mario.stanke@uni-greifswald.de),

# O. Keller, S. König, L. Gerischer, L. Romoth and Katharina Hoff.

# Please cite: Mario Stanke, Mark Diekhans, Robert Baertsch, David Haussler (2008),

# Using native and syntenically mapped cDNA alignments to improve de novo gene finding

# Bioinformatics 24: 637-644, doi 10.1093/bioinformatics/btn013

# No extrinsic information on sequences given.

# arabidopsis version. Using default transition matrix.

# We have hints for 0 sequences and for 0 of the sequences in the input set.

#

# ----- prediction on sequence number 1 (length = 13705, name = 2552694) -----

#

# Predicted genes for sequence number 1 on both strands

# start gene g3

2552694 AUGUSTUS gene 9169 13521 0.02 - . g3

2552694 AUGUSTUS transcript 9169 13521 0.02 - . g3.t1

2552694 AUGUSTUS tts 9169 9169 . - . transcript_id "g3.t1"; gene_id "g3";

2552694 AUGUSTUS exon 9169 9511 . - . transcript_id "g3.t1"; gene_id "g3";

2552694 AUGUSTUS stop_codon 9401 9403 . - 0 transcript_id "g3.t1"; gene_id "g3";

2552694 AUGUSTUS intron 9512 9806 0.65 - . transcript_id "g3.t1"; gene_id "g3";

2552694 AUGUSTUS intron 10080 10328 0.97 - . transcript_id "g3.t1"; gene_id "g3";

2552694 AUGUSTUS intron 10584 11039 0.97 - . transcript_id "g3.t1"; gene_id "g3";

2552694 AUGUSTUS intron 11179 11385 0.93 - . transcript_id "g3.t1"; gene_id "g3";

2552694 AUGUSTUS intron 11602 12122 0.5 - . transcript_id "g3.t1"; gene_id "g3";

2552694 AUGUSTUS intron 12505 12810 0.91 - . transcript_id "g3.t1"; gene_id "g3";

2552694 AUGUSTUS intron 12900 12986 0.59 - . transcript_id "g3.t1"; gene_id "g3";

2552694 AUGUSTUS intron 13118 13325 0.96 - . transcript_id "g3.t1"; gene_id "g3";

2552694 AUGUSTUS CDS 9401 9511 0.65 - 0 transcript_id "g3.t1"; gene_id "g3";

2552694 AUGUSTUS CDS 9807 10079 0.97 - 0 transcript_id "g3.t1"; gene_id "g3";

2552694 AUGUSTUS exon 9807 10079 . - . transcript_id "g3.t1"; gene_id "g3";

2552694 AUGUSTUS CDS 10329 10583 0.97 - 0 transcript_id "g3.t1"; gene_id "g3";

2552694 AUGUSTUS exon 10329 10583 . - . transcript_id "g3.t1"; gene_id "g3";

2552694 AUGUSTUS CDS 11040 11178 0.97 - 1 transcript_id "g3.t1"; gene_id "g3";

2552694 AUGUSTUS exon 11040 11178 . - . transcript_id "g3.t1"; gene_id "g3";

2552694 AUGUSTUS CDS 11386 11601 0.56 - 1 transcript_id "g3.t1"; gene_id "g3";

2552694 AUGUSTUS exon 11386 11601 . - . transcript_id "g3.t1"; gene_id "g3";

2552694 AUGUSTUS CDS 12123 12504 0.87 - 2 transcript_id "g3.t1"; gene_id "g3";

2552694 AUGUSTUS exon 12123 12504 . - . transcript_id "g3.t1"; gene_id "g3";

2552694 AUGUSTUS CDS 12811 12899 0.6 - 1 transcript_id "g3.t1"; gene_id "g3";

2552694 AUGUSTUS exon 12811 12899 . - . transcript_id "g3.t1"; gene_id "g3";

2552694 AUGUSTUS CDS 12987 13117 0.59 - 0 transcript_id "g3.t1"; gene_id "g3";

2552694 AUGUSTUS exon 12987 13117 . - . transcript_id "g3.t1"; gene_id "g3";

2552694 AUGUSTUS CDS 13326 13466 0.96 - 0 transcript_id "g3.t1"; gene_id "g3";

2552694 AUGUSTUS exon 13326 13521 . - . transcript_id "g3.t1"; gene_id "g3";

2552694 AUGUSTUS start_codon 13464 13466 . - 0 transcript_id "g3.t1"; gene_id "g3";

2552694 AUGUSTUS tss 13521 13521 . - . transcript_id "g3.t1"; gene_id "g3";

# coding sequence = [atgtctcccaggtcaggcgacctggtttgggtcggacccaggttagggttcgaccctggctgggtccaattgacccttg

# ggtccgacccaaaccaggttgcccgacctgagagacctagtcggggtcggacccaggccagggaggcgaagtacggtgatgatattgagaggatgaag

# acttatgtgaagggtcggcttgtcagagacacgatggatgatgatccacttgaaaggctcgagttcatcgatattgttcaacgattaggactgcactg

# ctacaataatatctttgacgatgatgtgtatgctacggctctcctgtttcgacttctaaggcaacacgggtataacctccaccaagaggtgtttaaga

# agtttatggatggtgagagtggttcctttagagaatcactgagtgaagatgtgaaggggatgttgagtttgtatgaagcttcttttcatggacggacg

# gacgaagctattgttgatgaagcaatgacattctctactgcatctttgagaaagaagactactttgacagtagaaactactagaatggctcaaatggt

# ggaacatgctttagacatgcctatccattggaggccaaacagattggaggctcgatggttcatcgatgtctacagtgaggagccatgcatggacgtaa

# ctttgctgcgattagccaaactggactataacatggtccaatcgtcgcaccaaaagaatgtcgctgagctaatacggtggtgggtcgggttaggttta

# aataagattaccttcgttcgagacaggttggtggagcattacttgtggagttcaattatggtttttgagacacaatataaagctcatcttatagccaa

# tgccaagatagcttccatggttactaccatagatgattgttacgacatatatggcacgttggacgaattggagatcttaacccaacttgttaaccgat

# gggacataaccgaagcggataggcttcctcatccgattagggtttgctttgtcgctctgttcaacaccaccaatgaaatcggactcgagttaatggtg

# gagcacggatataacttcattccatacttacataagatgtggataggtcaatgcaatgcatatatggaagaggcaaggtggtaccataatggaataaa

# gccaacatttaatgagtacctcaccaacggcatctgctctgtcggaacagtaatagggctattttgcacatttcttaccaccacaactgcaactgata

# tcaatgaagaaacactagattttatatctgagattcccacaatcatgcgctcctccaccgtgattgttcgactcaataacgacttagccacgtcatcg

# tatgaactagctagaggggacaacctcaaggctgtggaatgctacatagctgaaactggatgttcagaagaagctgctcggctgcatatccggaatct

# ggtgggggacacgtggaaggcgatgaatgaggctgctttaatcaaatacccattcaagggaccctttgtggaggcgtgcttcaaccttgctagggcat

# ctcaatgcttctaccagtacggagatggtcacggtcttcccaacaatgaaaccaaacagcatatcatgtccattctgcagtggtctgtagcagctgaa

# ccggctggttcagctgctgccatatggtctgcagcagctgaaccgaccggttcagctgctgcagcagtgctctgctgcagcagcagctaa]

# protein sequence = [MSPRSGDLVWVGPRLGFDPGWVQLTLGSDPNQVARPERPSRGRTQAREAKYGDDIERMKTYVKGRLVRDTMDDDPLER

# LEFIDIVQRLGLHCYNNIFDDDVYATALLFRLLRQHGYNLHQEVFKKFMDGESGSFRESLSEDVKGMLSLYEASFHGRTDEAIVDEAMTFSTASLRKK

# TTLTVETTRMAQMVEHALDMPIHWRPNRLEARWFIDVYSEEPCMDVTLLRLAKLDYNMVQSSHQKNVAELIRWWVGLGLNKITFVRDRLVEHYLWSSI

# MVFETQYKAHLIANAKIASMVTTIDDCYDIYGTLDELEILTQLVNRWDITEADRLPHPIRVCFVALFNTTNEIGLELMVEHGYNFIPYLHKMWIGQCN

# AYMEEARWYHNGIKPTFNEYLTNGICSVGTVIGLFCTFLTTTTATDINEETLDFISEIPTIMRSSTVIVRLNNDLATSSYELARGDNLKAVECYIAET

# GCSEEAARLHIRNLVGDTWKAMNEAALIKYPFKGPFVEACFNLARASQCFYQYGDGHGLPNNETKQHIMSILQWSVAAEPAGSAAAIWSAAAEPTGSA

# AAAVLCCSSS]

# end gene g3
