# Supplementary material for: Differential gene expression associated with a floral scent polymorphism in the evening primrose Oenothera harringtonii (Onagraceae)
Source: BMC Genomics. 2022 Feb 12;23:124. doi: 10.1186/s12864-022-08370-6 (PMC8840323; doi:10.1186/s12864-022-08370-6)
Supplement: Supplementary file 8 — Additional file 8. Alignment of the predicted full-length Oenothera harringtonii (R)-(−)-linalool synthase protein sequence with the Backhousia citriodora (R)-(−)-linalool synthase protein sequence and the merged predicted (R)-(−)-linalool synthase transcripts from the O. harringtonii reference transcriptome assembly. [file 12864_2022_8370_MOESM8_ESM.doc]

>BAG82825.1 linalool synthase [Backhousia citriodora]

MALPALFGSSLPSSIRHNQPSLFSFRHPRFCSSSSSASFSSQFILCASKTGDQEIVRRSANWQPSVWDYDYVQSLTVDYTEDKYTKQVQRLKEEVKGLFDREM---KQVAKLEFIDVVQRLGLGYHFKTEIKIALSSIHNNTEDAQLSNDLYAASLRFRLLRQYGCNVQQDVFQRFMN-KTGTFKESLNKDVKGILGLYEASFHGMEGETVLDEAWNFASKHL-KDLNLDEVPTNLASNVSHALDMPIHWRPNRLEARWFMDMYEKQQDLIPSLLRLAKLDFNIVQSIHRKEVSNLARWWVELGANKMTFFRDRLVESYFWSCILVFEPQYTDFRELNTRIACMATLIDDVYDIYGTPEELELLTDFILRWDITDIDKLPPTIRNGFMALYNTTNKVGYRTMTKRGINPIPYLRKLWGDECKADMKEVHWFNNGIKPTLKEYMDVAVDSIGGLILLLNSYFLTT--DYLTEEGLNYVSKIPSVMHSSAQIFRFNDDLSTSSHELARGDNSKALECYMNETGASEEIAREHIRHLVRETWKKMNKEVFEDYPFSGFGPFLSACLNLARASHCFYEYGDGYGLPDHQTRDHLASTIFESVSLD-----------------------------

>g3.t1 [Predicted full-length (R)-(-)-linalool synthase gene from Oenothera harringtonii]

----------------------MSPRSGDLVWVGPRLGFDPGWVQLTLGSDPNQVARPERPSRGR-----------TQAREAKYGDDIERMKTYVKGRLVRDTMDDDPLERLEFIDIVQRLGLHCY-----------------NNIFDDDVYATALLFRLLRQHGYNLHQEVFKKFMDGESGSFRESLSEDVKGMLSLYEASFHGRTDEAIVDEAMTFSTASLRKKTTLTVETTRMAQMVEHALDMPIHWRPNRLEARWFIDVYSEEPCMDVTLLRLAKLDYNMVQSSHQKNVAELIRWWVGLGLNKITFVRDRLVEHYLWSSIMVFETQYKAHLIANAKIASMVTTIDDCYDIYGTLDELEILTQLVNRWDITEADRLPHPIRVCFVALFNTTNEIGLELMVEHGYNFIPYLHKMWIGQCNAYMEEARWYHNGIKPTFNEYLTNGICSVGTVIGLFCTFLTTTTATDINEETLDFISEIPTIMRSSTVIVRLNNDLATSSYELARGDNLKAVECYIAETGCSEEAARLHIRNLVGDTWKAMNEAALIKYPFK--GPFVEACFNLARASQCFYQYGDGHGLPNNETKQHIMSILQWSVAAEPAGSAAAIWSAAAEPTGSAAAAVLCCSSS

>OhRLS [merged transcripts from Oenothera harringtonii reference transcriptome assembly]

-----------------------------------------------------------------------------------------------------------------------------------------------------------------------------------------------------------------------------------------------------------------------------MDVTLLRLAKLDYNMVQSSHQKNVVELKRWWTGLGLGKMSFFRDRLMEHYLWIALTVINPQYKAHLIATSKIACMITTIDDCYDVYGTLEEVELLTEFVQRWDITEIDRLPPAIRSCFLALYNTTNEIGLELMVATGYNFIPYLQKMWANQCKAYLREAKWYHNGVKPTFKEYLNNGVTTVGGFIMLLSCFLTTA--NNINEETLDYVSKIPAIMRCSSTILRLNNDLGSSSYELARGDNLKAIECYMAETGSSEGTAREHVQNIVRETWKTMNEVAFISYPFK--GPFVDACINFARASQCFDQHGNCHGLPSDVMNSHIMSILVQSVPMDHV---------------------------
