# Supplementary material for: Differential gene expression associated with a floral scent polymorphism in the evening primrose Oenothera harringtonii (Onagraceae)
Source: BMC Genomics. 2022 Feb 12;23:124. doi: 10.1186/s12864-022-08370-6 (PMC8840323; doi:10.1186/s12864-022-08370-6)

Supplemental Methods, Flower Part Emissions

Plant material:

Oenothera harringtonii plants from a linalool producing (+) population (FLO = Florence, Colorado, USA) and others from a non-linalool producing (-) population (DC = David Canyon, Colorado, USA) were cultivated under greenhouse conditions at Cornell University, Ithaca NY, as described in the main text Methods. Rosette plants were transferred to 70% sand: 30% soil mix in clay pots in February 2021 and initiated blooming in early April.

Experimental treatments:

We sought to determine whether R-(-)-linalool and other volatile compounds are emitted globally by all floral tissues or show specific tissue-specific emission patterns. This is often done on an exploratory basis, using floral headspace equilibration and absorption techniques, such as solid phase microextraction (SPME), to collect scent from dissected floral tissues in small headspace bags or beakers, as was done in a previous study revealing nectar spur-specific S-(+)-linalool production in *Penstemon digitalis* (Burdon et al. 2015). Instead, we used dynamic headspace collection from living flowers, from which specific floral tissues had been excised, providing quantitative data on emission rates per floral unit or fresh mass during the first hour of anthesis. This approach was used effectively by Pichersky et al. (1994) to compare S-(+)-linalool emissions from petals and other floral organs in *Clarkia breweri* (Onagraceae).

At anthesis (bud break), we selected 2-3 newly opened flowers on FLO and DC plants, whenever possible having at least one positive control (whole, intact flower) per plant to compare with a modified flower. Treatments referred to as “petals” were flowers whose anthers and style were removed using sharp, watchmaker’s forceps, whereas the “sex organs” treatment consisted of flowers whose petals had been removed in a similar fashion. In no case did a treated flower wilt or lose turgor pressure during the ensuing 1 hr headspace collection. We collected four replicates of each floral treatment x both DC and FLO populations, from which an additional two samples each were collected from unopened flower buds (negative control) one day before anthesis, yielding a total of 28 headspace samples. Fresh floral masses were measured to the 0.0001 g for calculations of mass-standardized emission rates, using a laboratory digital balance.

Sample collection and processing:

Single flowers (controls and treatments) were covered with nylon resin “oven bags”, cut and sealed to standardized dimensions suitable for flowers of *O. harringtonii* (12 x 15 cm). Scent traps constructed by sandwiching 10 mg of SuperQ (80-100 mesh size) absorbent particles between plugs of silanized quartz wool in modified glass Pasteur pipettes were enclosed within floral headspace bags, cinched with supermarket twist-ties. Traps were connected via 3cm lengths of Tygon tubing to individual 9V battery operated vacuum pumps (PAS-500, Spectrex, Inc.), which pulled scent-laden air through the traps at 200 ml air/min. Pumps were turned on shortly after the time of anthesis (20:00-20:15 hrs) and headspace samples were collected for one hr. Volatiles were eluted by washing scent traps with 300 uL of GC-MS grade hexane solvent into 1.5 mL amber glass autoinjector vials fitted with shell-style glass inserts and capped with Teflon-sided septa, then were stored at -20C until analysis by gas chromatography-mass spectrometry (GC-MS). On the day of analysis, glass inserts were positioned below a gentle flow of N2

gas to concentrate eluted samples from 300 to 50 uL total volume, to which we added 5 uL of 0.03% toluene in hexane as an internal standard.

Sample analysis by GC-MS:

Samples were analyzed by injecting 1 uL aliquots into a Shimadzu Scientific Instruments GC2010+ GC-MS. Compounds were separated on a polar GC column (Stabilwax, 30 m length, 0.25 mm internal diameter, 25 um film thickness) with the following program: split injection (10:1 ratio) at 240C, oven program hold 3 min at 40C, increase at 10C/min to 240C, then hold 3 min (total run time 26 min). This program allowed the resolution of all floral volatile peaks to baseline, and peak areas were integrated manually using the Shimadzu GCMSsolutions software version 4.45. Volatile peaks were quantitated using the toluene internal standard (23.4 ng in 1 uL), yielding emissions in toluene equivalents per flower and per fresh floral mass. Data were collected for the full sets of volatile compounds present in control (whole) flowers for DC and FLO plants, ignoring small peaks of typical wound volatiles (e.g. (Z)-3-hexene-1-ol, (Z)-3-hexenyl acetate) present in “petal” or “sex organ” treatments.

References:

Burdon, R. C., Raguso, R. A., Kessler, A., & Parachnowitsch, A. L. (2015). Spatiotemporal floral scent variation of *Penstemon digitalis*. *Journal of Chemical Ecology*, *41*(7), 641-650.

Pichersky, E., Raguso, R. A., Lewinsohn, E., & Croteau, R. (1994). Floral scent production in *Clarkia* (Onagraceae)(I. Localization and developmental modulation of monoterpene emission and linalool synthase activity). *Plant Physiology*, *106*(4), 1533-1540.

Supplemental Material: Results and Discussion

Total ion chromatograms (GC-MS) of floral scent emitted from living, intact flowers of *Oenothera harringtonii* during the first hour of evening anthesis (c. 20:00-21:00 hrs), April 2021. Volatile compounds have been separated on a polar GC-column (stabilwax). GC traces compare unopened bud (lowest, blue), floral tube with petals excised (sex organs, green), floral tube with sex organs removed (petals, brown) and whole, uncut flowers (black) of the linalool-producing population from Florence, Colorado, USA (FLO). T represents the internal standard, 23.4 ng of toluene in hexane. Peak numbers refer to specific compounds summarized in the table below. R-(-)-linalool is peak #8. Peak areas show that volatile emissions from petals are comparable in chemical composition and emission rates to those of the whole flower control, whereas emissions from sex organs are substantially weaker and are missing minor components (ocimene and farnesol derivatives) of the full volatile blend.

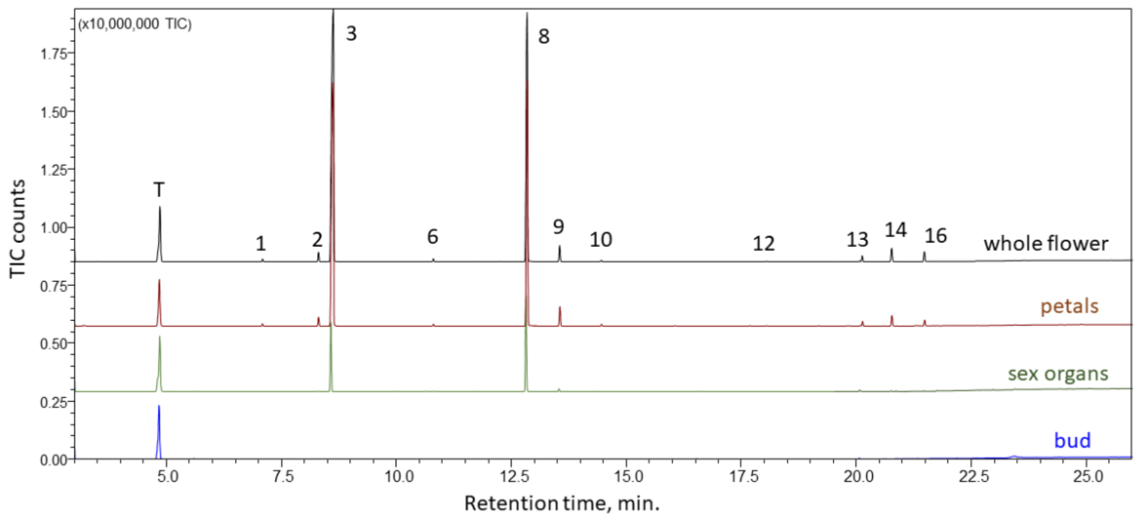

Supplement: Supplementary file 9 — Additional file 9. GC-MS evidence that volatile emissions from petals ((R)-(−)-linalool in particular) are comparable in chemical composition and emission rates to those of the whole flower control, whereas emissions from sex organs are substantially weaker and are missing minor components (ocimene and farnesol derivatives) of the full volatile blend. [file 12864_2022_8370_MOESM9_ESM.pdf]
